# A systematic review and meta-analysis of diabetes and risk of physical disability and functional impairment - protocol

**DOI:** 10.1186/2046-4053-1-47

**Published:** 2012-10-17

**Authors:** Evelyn Wong, Kathryn Backholer, Jessica Harding, Emma Gearon, Christopher Stevenson, Rosanne Freak-Poli, Anna Peeters

**Affiliations:** 1 Department of Epidemiology & Preventive Medicine, Faculty of Medicine, Nursing & Health Sciences, School of Public Health and Preventive Medicine, Monash University, The Alfred Centre, Alfred Hospital, Commercial Road, Melbourne, VIC, 3004, Australia; 2 BakerIDI Heart and Diabetes Institute, 99 Commercial Road, Melbourne, VIC, 3004, Australia; 3 School of Health and Social Development, Faculty of Health, Deakin University, 221 Burwood Highway, Burwood, VIC, 3125, Australia

**Keywords:** Diabetes, Impaired glucose tolerance, Physical disability, Functional limitation, Mobility limitation, Systematic review, Meta-analyses, Observational studies, Activities of daily living

## Abstract

**Background:**

Diabetes and increased age are known risk factors for physical disability. With the increasing prevalence of diabetes within our aging population, the future burden of disability is expected to increase. To date, there has not been a pooled estimate of the risk for disability associated with diabetes or its precursor states, impaired glucose tolerance and impaired fasting glucose. We aim to conduct a systematic review and meta-analysis of the association between prediabetes and diabetes with disability, and quantify the risk of association.

**Methods/design:**

We will search for relevant studies in Medline via Pubmed, Embase, Cochrane library and Cumulative Index to Nursing and Allied Health Literature (CINAHL), as well as scan reference lists from relevant reviews and publications included in our review. We will review all publications that include studies on human adults (18 years and older) where information is included on diabetes status and at least one measure of disability (Activities of Daily Living (ADL), Instrumental ADL (IADL) or functional/mobility limitation), and where a risk association is available for the relationship between diabetes and/or prediabetes with disability, with reference to those without diabetes.

We will further conduct a meta-analysis to pool estimates of the risk of disability associated with prediabetes and diabetes. Sensitivity analysis will be conducted to assess for publication bias and study quality.Findings from this systematic review and meta-analysis will be widely disseminated through discussions with stake-holders, publication in a peer-reviewed journal and conference presentation.

## Background

With increasingly aging populations and increasing prevalence of diabetes, there are fears that the gains in healthy life expectancy that have been made may soon be reversed. Estimations of the impact of these trends on the burden of ill health and disability in old age depend on accurate estimations of the associated risks. Although it is clear that diabetes is associated with an increased risk of physical disability, the magnitude of this association is unclear.

Diabetes mellitus is a metabolic condition characterized by hyperglycemia due to defects in insulin action or secretion or both, resulting in end-organ damages such as ischemic heart disease, chronic kidney disease, strokes, peripheral neuropathy and peripheral vascular disease [1]. Diabetes may lead to disability either via one of the above mentioned pathways or independently [2].

Physical disability is defined by ‘long term limitations in major activities of daily life’ [3]. There are various measures of this limitation including limitations to activities such as feeding, washing, dressing, transferring in and out of a chair or bed, and getting to and using a toilet, all of which can be categorized as limitations to self-care activities of daily living (ADL). Mobility limitations are also used to measure disability and these can be self-reported limitations to various walking tasks or measured functions such as walking-speed.

Estimates of the association between diabetes and disability vary. A study of a prospective cohort of elderly Mexican-Americans free of disability at baseline (1993–1994) reported that participants with diabetes were twice as likely to report development of any limitation in lower body disability during a seven-year follow-up (hazard ratio (HR) 2.05, 95% confidence interval (CI) 1.58-2.67) compared to those without diabetes [4]. Similarly, a prospective study of elderly women, commenced in 1986, reported a HR of 1.42 (95% CI 1.23-1.65) [5]. In contrast, a cross-sectional study in Taiwan, revealed that for both men and women the risk of disability among those with or without diabetes did not significantly differ for men (odds ratio (OR) 1.1 (95% CI 0.6-2.2)) or women (OR 1.7 (95% CI 0.7-4.2)) [6]. In a similar population cross-sectional survey in Hong Kong, Chau *et al*. reported an OR of 1.7 (95% CI 1.51-1.8) for disability in those undergoing treatment for diabetes when compared to those without diabetes [7].

Variations in these risk estimates are likely to reflect differences in the measurement of disability, the populations examined, baseline age of participants in the study, follow-up time, diabetes duration**,** method of ascertaining diabetes and disability status, as well as confounders adjusted for in multivariable regression models. Measurements of disability in various studies to date have included self-reported limitations to ADLs [4,7,8], instrumental ADLs [5,8] or mobility tasks [4,5] and measured mobility limitation such as a timed 8-foot walk [4], lower extremity function, balance [5], and hand function [9]. Several of these studies have included only women.

Other factors which appear to influence the relationship between diabetes and disability include age, sex, education, smoking, lack of physical activity, ethnicity, obesity and other comorbidities including depression [2,10,11].‘Prediabetes’ defined by impaired glucose tolerance (IGT) or impaired fasting glucose (IFG) is an intermediate state where glucose levels do not meet the criteria for a diagnosis of diabetes but are above normal accepted levels [1]. The risk of disability associated with prediabetes is less studied. In a small cohort (n=88), an increased risk of disability was observed for those having diabetes for over 15 years and increased glycosylated hemoglobin (HbA1C) but this was not the case for short-term or newly diagnosed diabetes or those with IGT [9].

We aim to conduct a systematic review of the association between diabetes, prediabetes and physical disability and quantify this relationship using a meta-analysis. If sufficient data permit, we further aim to examine the moderating effect of sex, age and duration of diabetes on the above mentioned associations.

## Methods/Design

### Literature search strategy

Our search strategy, selection of publications and the reporting of results for the review will be conducted in accordance with the PRISMA guidelines [12]. We will search the following databases: Ovid Medline, Embase, Cochrane library and Cumulative Index to Nursing and Allied Health Literature (CINAHL). We will further scan reference lists in relevant reviews and publications retrieved for the purpose of our review. There will be no initial limit on the date of publication. We will search for publications with medical subject headings (MeSH) and keywords in title, abstract and text for diabetes and disability terms. The diabetes MeSH and keyword terms will include ‘diabetes’, ‘glucose intolerance’, combined with the operator ‘OR’. The disability MeSH terms will include ‘activities of daily living’ and ‘disabled persons’ and the disability keywords will comprise ‘disabl*’, ‘disabiliti*’, ‘limit*’ and ‘impair*’, where * indicates an open ending to the word. Where terms are exploded, this will be recorded. All disability MeSH and keyword terms will be combined with the operator ‘OR’. Following the separate searches for diabetes and disability terms, the search will then be combined with the operator ‘AND’.

We will further limit our search to exclude all publications on animals only and on humans younger than 18 years of age. A limit will also be placed on type of studies to include only case-control studies, cohort studies, cross-sectional studies and clinical trials.

### Inclusion criteria

For inclusion, publications must present studies of human adults (18 years of age and older) where information is included on diabetes status and at least one measure of disability (ADL or functional/mobility limitation), and where a risk association is reported for the relationship between diabetes or prediabetes and disability compared to no diabetes and disability. If there is more than one publication from the same study, we will only include the latest publication.

### Types of studies

This review will include case-control, cross-sectional and cohort studies as well as clinical trials.

### Types of participants

Participants will be adults aged 18 years and older with recorded status of diabetes (has diabetes, no diabetes or has ‘prediabetes’) or impaired glucose tolerance and physical disability or mobility limitation.

For the purpose of this review, there will be no limit on the type of diabetes. Diabetes can be classified as gestational diabetes, Type 1 diabetes or Type 2 diabetes depending on etiology. Diagnosis of diabetes can either be made by physician or defined by available measured fasting glucose or glucose tolerance test or self-reported.

Prediabetes is defined by having IFG (fasting plasma glucose (FPG) levels ≥100 mg/dl (5.6 mmol/L) but <126 mg/dl (7.0 mmol/L)) or IGT (2-hour oral glucose tolerance test (OGTT) of ≥140 mg/dl (7.8 mmol/L) but <200 mg/dl (11.1 mmol/L)) [1].

### Types of outcome measures

Our outcome measure of interest will be disability defined by limitation in one or more ADLs or in mobility. If possible, we will have three outcome measures: (1) limitations to basic ADLs (such as bathing, dressing, eating, getting in or out of bed, toileting); (2) limitations to instrumental ADL’s (such as housework, shopping); and (3) limitations to mobility (such as walking a certain distance and going up and down stairs).

Once the search has been conducted, at least two authors will cross check retrieved articles and make independent judgments as to which publications should be included in the study. First, studies will be selected if the title of the study is relevant. Next, the abstract will be assessed to determine if the study satisfies the inclusion criteria. If it is unclear from the abstract as to whether selection criteria are met, the full article will be scanned. Any discrepancy in study inclusion will be discussed with a third or fourth author. Once the appropriate articles have been chosen for further analysis, two or three authors will be involved in each article’s independent assessment and extraction of data. Further studies may be excluded as a result of not meeting the inclusion criteria. Further studies may be included through searching reference lists in publications selected for the review. All included studies will be read in detail and relevant information extracted.

### Data extraction

From the selected publications, we will extract where available: type of study design; year of study; length of follow-up; person-years of follow-up; sample size; proportion of men and women; age; population characteristics (such as general population, health professionals, ethnic composition); method used to ascertain diabetes status; number of cases with disability; method used to ascertain disability; incidence rate of disability; list of factors adjusted for; relative risk or equivalent associated with pre-diabetes or diabetes; and disability. Effect sizes will be extracted from multi-variable regression models that adjusted for the most comprehensive set of plausible confounders. If effect sizes are only available from unadjusted models, these effect sizes will be extracted and these studies will be noted in quality analysis.

Wherever possible, adjusted relative risk (RR) or equivalent and associated 95% CI will be extracted directly from studies. For studies that present RR for subgroups (for example, sex) the data for each individual subgroup will be additionally extracted. Authors will be contacted via email for any missing relevant information.

### Data analysis

As disability is not rare, we will not be able to treat all HRs, RRs and ORs as the same. If the data permit we will either convert the effect size to the most common form of estimate, or we will group studies by the type of effect size and pool these different effect size estimates.

To pool relative effects, we will be using the Generic Inverse Variance (GIV) method in Review Manager (RevMan 5.1.7) software (The Nordic Cochrane Centre, Copenhagen, Denmark). Log of the ratios of effect (RR, OR or HR) and its standard error (SE) will be calculated and entered into RevMan.

We plan to analyze the magnitude of association by study design and therefore, we will pool point estimates from groups of studies with the same study design. Where data permit, we will further conduct a subgroup analysis by age (<65 years of age versus ≥65 years of age), sex and diabetes duration (<15 years since diagnosis versus ≥15 years since diagnosis). In these subgroup analyses, significant differences in pooled effect size between subgroups will be tested using Revman 5.1.7.

### Assessment of heterogeneity and quality

A random effect model will be used as a conservative approach as data is expected to vary across studies. Heterogeneity will be tested using the chi-squared statistic, where a *P* value less than 0.1 will be regarded as significant heterogeneity [13]. Other assessments of heterogeneity will include I^2^ (I^2^ ≥75% equates to high heterogeneity [14]) and visual inspection of the forest plot for inconsistencies in effect sizes and their confidence intervals. After using the Newcastle-Ottawa Scale for study quality [15], a separate analysis will be performed on studies with the highest level of quality. A high-quality publication will be defined as one that is a prospective cohort study with disability-free participants at baseline, measured glucose tests to diagnose diabetes or physician-diagnosed diabetes, and regression models adjusting for major covariates. Where mobility limitation is a reported outcome, measured mobility limitation will be considered as an additional quality criterion.

If data permit, a metaregression will be conducted to analyze for sources of heterogeneity.

### Assessment of reporting bias

Symmetry of funnel plots will be used to assess for publication or selective reporting bias.

## Discussion

This systematic review and meta-analysis will provide an updated and quantitative estimate of the risk of disability associated with prediabetes and diabetes compared to those without. This pooled estimate will take into account factors which may confound or modify the relationship such as sex, socio-economic position, and duration of diabetes, and will indicate the role of such factors in the variability of reported estimates. This review will include a wide number of study designs with a subgroup analysis to study the effects of study design and study quality on the estimate the risk association between diabetes and disability.

The findings from the review will be widely disseminated through discussions with stake-holders, publication in a peer-reviewed journal and a conference presentation. The pooled estimate will guide clinicians and policy makers in informing patients and governments about the risk of disability associated with diabetes, and will likely aid the promotion of disability prevention measures. It will further provide an estimate that can be used in future projections of disability prevalence in our aging population. A systematic review on diabetes and disability will bring to light knowledge gaps in the area and offer directions for future research.

## Abbreviations

ADL: activities of daily living; IADL: instrumental activities of daily living; CINAHL: Cumulative Index to Nursing and Allied Health Literature; FPG: fasting plasma glucose; HR: hazards ratio; IFG: impaired fasting glucose; IGT: impaired glucose tolerance; MeSH: medical subject heading; OGTT: oral glucose tolerance test; OR: odds ratio; PRISMA: preferred reporting items for systematic reviews and meta-analyses; RR: risk ratio.

## Competing interests

The authors declare that they have no competing interests.

## Authors’ contributions

EW was responsible for the- formulation of research question, design of protocol, drafting the manuscript and was also responsible for manuscript submission and responding to reviewer comments. KB -assisted with the formulation of research question, design of protocol and commented on manuscript drafts. JH, EG, CS and RFP -commented on manuscript drafts. AP -assisted with formulation of research question, design of protocol and commented on manuscript drafts. All authors read and approved the final manuscript.
